# Wide-incident-angle, polarization-independent broadband-absorption metastructure without external resistive elements by using a trapezoidal structure

**DOI:** 10.1038/s41598-024-60171-x

**Published:** 2024-05-03

**Authors:** Thanh Son Pham, Haiyu Zheng, Liangyao Chen, Bui Xuan Khuyen, YoungPak Lee

**Affiliations:** 1 Department of Physics and Quantum Photonic Science Research Center, Hanyang University, Seoul, 04763 Korea; 2 Alpha ADT, No.1202, 51-9, Dongtan Advanced Industrial, Hwaseong, 18469 Korea; 3 Department of Optical Science and Engineering, Fudan University, Shanghai, 200433 China; 4 Institute of Materials Science, Vietnam Academy of Science and Technology, 18 Hoang Quoc Viet, Cau Giay, Hanoi, 100000 Vietnam

**Keywords:** Applied physics, Optical physics

## Abstract

The absorption of electromagnetic waves in a broadband frequency range with polarization insensitivity and incidence-angle independence is greatly needed in modern technology applications. Many structures based on metamaterials have been suggested for addressing these requirements; these structures were complex multilayer structures or used special materials or external electric components, such as resistive ones. In this paper, we present a metasurface structure that was fabricated simply by employing the standard printed-circuit-board technique but provides a high absorption above 90% in a broadband frequency range from 12.35 to 14.65 GHz. The metasurface consisted of structural unit cells of 4 symmetric substructures assembled with a metallic bar pattern, which induced broadband absorption by using a planar resistive interaction in the pattern without a real resistive component. The analysis, simulation, and measurement results showed that the metasurface was also polarization insensitive and still maintained an absorption above 90% at incident angles up to 45°. The suggested metasurface plays a role in the fundamental design and can also be used to design absorbers at different frequency ranges. Furthermore, further enhancement of the absorption performance is achieved by improved design and fabrication.

## Introduction

In the last decade, there has been significant interest in the utilization of metamaterials for manipulating electromagnetic (EM) waves^1–3^. These metamaterials possess unique properties that do not occur naturally, making them highly desirable for a wide range of applications spanning from kHz to THz frequencies and even encompassing light waves^4–7^. Metamaterials are artificially engineered materials that exhibit novel and highly advantageous characteristics^8^. The development of metamaterials for controlling EM waves involves various approaches, including two-dimensional (2D) and three-dimensional (3D) fabrication techniques, as well as the utilization of resistors or other electric components^9–11^. Each approach has its own set of advantages and disadvantages so that they can be applied to different scenarios^12^.

Metamaterials present unique and highly advantageous properties for absorbing EM waves with minimum reflectivity^13^. This characteristic was achieved by obtaining the impedance-matching conditions between the metamaterial structure and surrounding environment, enabling the efficient dissipation of all the incident energy^14^. As a result, metamaterial absorbers have significant potential for a wide range of applications, including military radar, information security, stealth technology, sensing, and energy harvesting^15–19^. Since Landy et al. demonstrated the absorption properties of metamaterials for EM waves in 2008^20^, numerous metamaterial-absorber structures have been developed and employed. Initially, these structures exhibited nearly perfect absorption peaks at specific frequencies, which were subsequently extended to multi-frequency absorption^21–23^. Various configurations of the unit cell have been suggested, including cut-wire pairs, rings, split rings, and square rings^24–26^.

The excellent absorption capability of a metamaterial at one or multiple resonance peaks is accompanied with several limitations when applied to practical systems. Consequently, there is a demand for designing a metamaterial structure that exhibits high absorption of EM waves across a broad frequency spectrum^27,28^. As a result, extensive studies have been conducted to achieve high absorption over a wide range of frequencies by using metamaterials^29^. To achieve high absorption across a wide range of frequencies by employing metamaterials, various multi-layer structures have been introduced^30^. The implementation of multi-layered metamaterial structures has been observed, wherein each layer was relevant to the absorption of EM waves at a specific frequency among a wide range of frequencies, leading to a broad absorption band^31^. Furthermore, a metamaterial structure with external resistors was also employed, where the entered energy in the structure was dissipated at the resistors, and the impedance-matching conditions were combined to achieve a wide absorption band^32^. However, these structures are often complex, require significant effort in manufacturing and are prone to damage. The development of a simple 2D metamaterial structure with high and broad absorption capability devoid of external electric components or special materials remains a remarkable challenge.

This work proposes a 2D metamaterial structure called a metasurface, which exhibits more than 90% absorption of EM waves in a broad frequency range of 12.35 to 14.65 GHz. The metasurface was designed on the front of an FR-4 substrate with a typical thickness of 2 mm, while a seamless copper layer is placed at the back to prevent the entered EM waves from escaping and obtain high absorption for the structure. An external electric component such as a resistor or a special material was not employed. The metasurface was fabricated entirely by using simple printed-circuit-board (PCB) technology without requiring additional steps such as incorporating resistors or high-absorption layers. Investigations on the dependence of the absorption properties on the polarization and incident angle of EM incident waves were also conducted to verify that the designed structure was practical for these applications. The results show that a high absorption above 90% is maintained over a wide frequency range. This metamaterial structure is promising for use in various defense and civilian applications, such as Ku-band spectral imaging, radar stealth, satellite communication, radar surveillance and thermal radiometry^33–36^.

## Results

### Design and analysis

**Figure 1:**
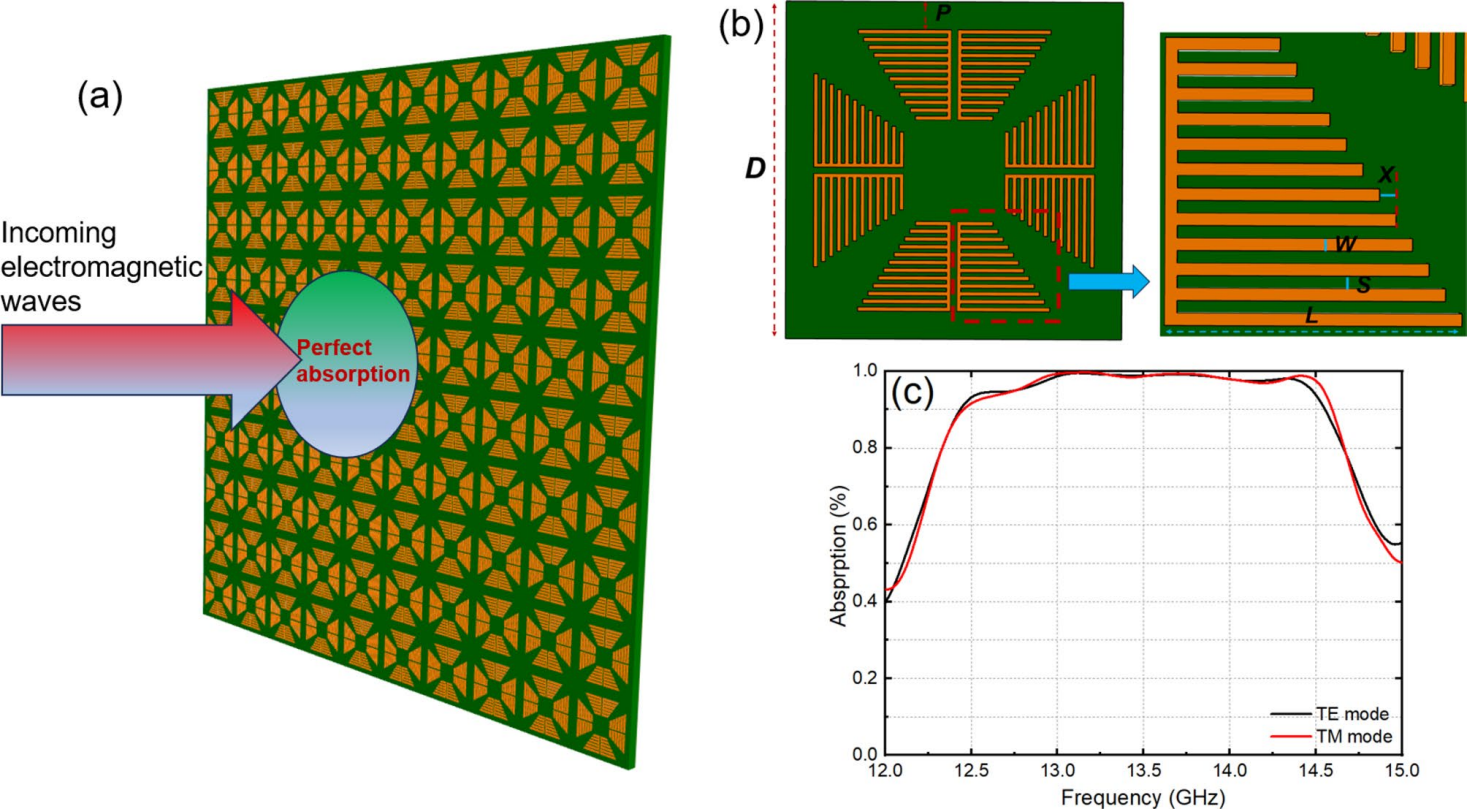
(**a**) Schematic illustration of the broadband-absorption metasurface, (**b**) the unit cell, and (**c**) high absorption in both the TE and TM modes.

It is acknowledged that metamaterial structures have the ability to absorb EM waves at the resonant frequency of the unit cell. In this way, structures that absorb multiple frequency peaks can be constructed by employing the corresponding multiple structures. While the utilization of conventional resonance structures with stacked resonance regions to create a broad absorption band has been explored, such structures were typically 3D or relied on specialized materials or external components^9,13,31^. On the other hand, Fig. 1a presents a proposed metasurface constructed, based on a 2D metamaterial. Notably, this metasurface offers the advantage of achieving broadband absorption without the need for specialized components or materials. The metasurface, illustrated in Fig. 1a, is composed of a 2D array of metamaterial unit cells, as shown in Fig. 1b. Each unit cell is characterized by a 4-sided symmetrical structure that comprises metallic (Cu) bars arranged in a trapezoidal shape, which adhere to an FR-4 substrate with a dielectric constant of ɛ = 4.3, a loss tangent of tan(*δ*) = 0.025, and a thickness of 2 mm. The unit cell is square-shaped and exhibits a period of *D* = 13 mm. In particular, each branch of the trapezoid is composed of metallic bars with decreasing lengths from the outermost bar with *L* = 3.5 mm, with a decreasing step of *X* = 0.2 mm. The width of the Cu bars is *W* = 0.15 mm, and the distance between the bars is *S* = 0.15 mm. Based on the unit-cell parameters specified above, the metasurface can absorb EM waves in a broad frequency range for both transverse-electric (TE) and transverse-magnetic (TM) polarizations, as depicted in Fig. 1c. The absorption of the metasurface exceeds 90% in the frequency range of 12.40 to 14.52 GHz. Specifically, the region of high absorption is characterized by a promoted degree of smoothness, and the majority of the absorption bands show an absorption level even exceeding 95%. These absorption results are particularly noteworthy since they were achieved through the use of a 2D metamaterial structure that did not rely on special materials or external electric components.

**Figure 2:**
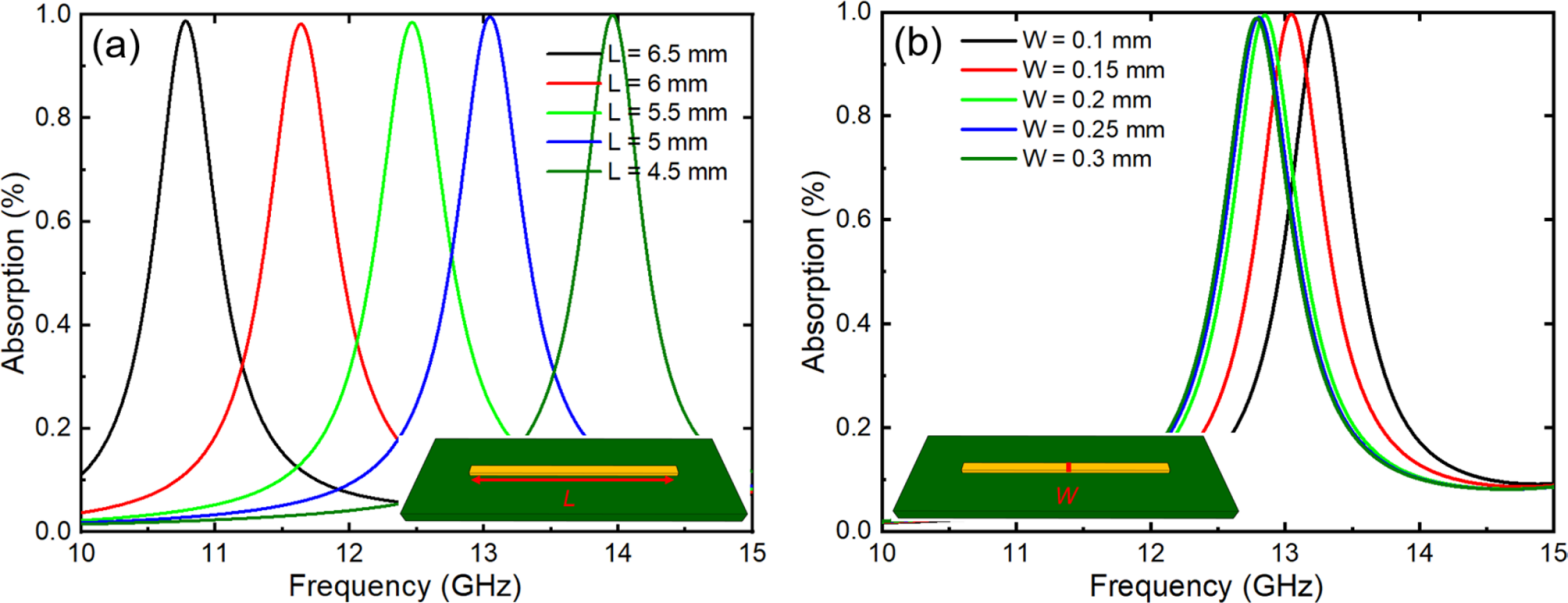
(**a**) Absorption of a single bar of various lengths (*L*). (**b**) A single bar with various strip widths (*W*).

To obtain a comprehensive understanding of the absorption properties of metasurfaces, we first examined the fundamental component of the designed metamaterial absorber: the metallic cut wire. This component is also referred to as a single bar below. Figure 2a shows the simulation results for the absorption spectrum of a single bar with a length *L* ranging from 6.5 to 4.5 mm on an FR-4 substrate with a thickness of 2 mm and a dielectric constant of 4.3. The results demonstrate that a single bar exhibits an absorption peak exceeding 99% in the frequency range of 10.8 to 13.9 GHz as the length decreases gradually from 6.5 to 4.5 mm. The resonance frequency of a single bar can be calculated by using the formula $f_{mn} = \frac{{c\sqrt {m^{2} + n^{2} } }}{{2L\sqrt {\varepsilon_{eff} } }}$, where *m* and *n* are integers (0, 1, 2,…) and $m^{2} + n^{2} \ne 0$, respectively, and *ɛ*_*eff*_ is the effective dielectric constant^24^. According to the above equation, the resonance frequency is inversely proportional to the length (*L*) of a single bar. For the major axis of a single bar, *m* and *n* correspond to the number of harmonics for the *y-* and *x*-axes, respectively. The resonance peak values in Fig. 2a are from the cases where *m* = 1 and *n* = 0. In addition, we investigated the impact of the single-bar strip width (*W*) on the intensity and peak frequency of the absorption spectrum. Figure 2b displays the absorption spectrum of a single bar with a length of 5 mm and a strip width varying from 0.1 to 0.3 mm. The results indicate that within the changing range of strip width, a single bar maintains an absorption above 99% at the resonance frequency. However, as the strip width increases, the absorption peak undergoes a small redshift.

**Figure 3:**
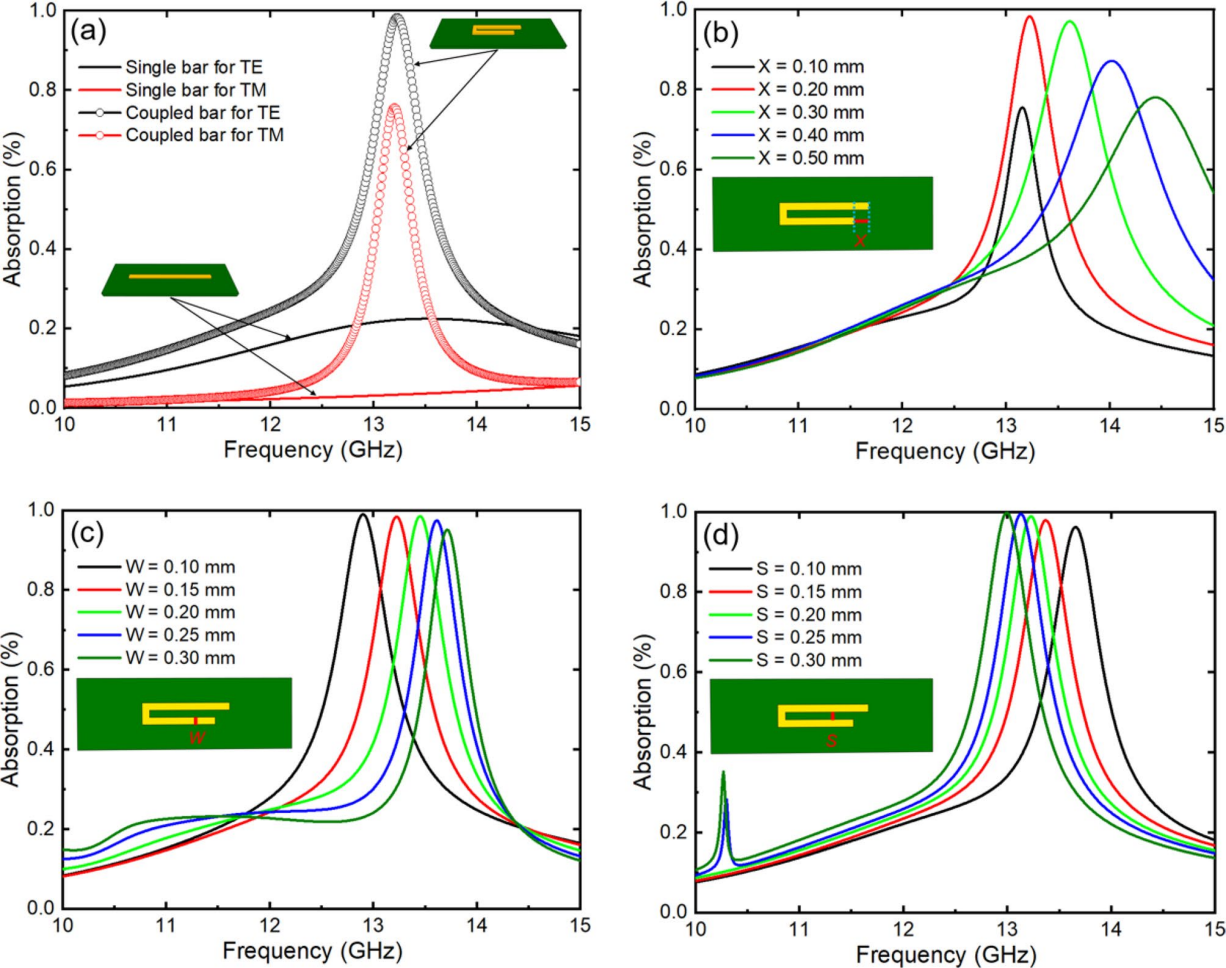
(**a**) Comparison of the absorption between single and coupled-bars; (**b**–**d**) absorption of a coupled-bar with various parameters: length difference (*X*), strip width (*W*), and spacing (*S*), respectively.

A single bar can provide nearly perfect absorption and easily control the absorption frequency but is highly sensitive to the polarization of the incident wave. When the incident polarization angle is 90°, the absorption response of a single bar is negligible. Therefore, we propose a coupled-bar configuration that works in both the TE and TM modes. Moreover, the frequency response of the coupled-bar can be estimated as the sum of the effective lengths of the constituent bars. This coupled-bar is composed of two single bars with different lengths by *X* = 0.2 mm, and the two single bars are combined by a connecting bar with a direction perpendicular to them. This allows us to have the resonant frequencies of smaller coupled-bars lower than those of single bars. Figure 3a shows a comparison of the absorption spectra between the single- and coupled-bar configurations for the TE and TM incident waves. In both configurations, the lengths of the single bar and the longer branch of the coupled-bar are set to 3.5 mm. For the single-bar case, the resonance frequency, corresponding to a length of 3.5 mm, is higher than the monitored frequency range of 10 to 15 GHz, resulting in the absence of a resonance peak in this frequency range. However, we still observe an absorption value of approximately 20% for the TE mode, while for the TM mode, the absorption value is close to 0. In contrast, the coupled-bar configuration exhibits an absorption peak in both the TE and TM modes at a frequency of 13.2 GHz, with absorption values of 99% and 76%, respectively.

Next, the absorption spectral properties of the coupled-bar configuration with varying shape parameters were investigated. Figure 3b shows the absorption spectra of the coupled-bars as the length difference (*X*) between the bars changes from 0.1 to 0.5 mm. The results reveal that the absorption spectrum reaches a maximum value of 99% or 97% for a length difference of 0.2 or 0.3 mm, respectively. However, for the other values of *X*, the absorption is reduced to less than 90%. Figure 3c shows the absorption spectra of coupled-bars with different strip widths (*W*) ranging from 0.1 to 0.3 mm. The absorption peaks are all above 90% and reach even 99% when *W* = 0.1, 0.15, and 0.2 mm. Furthermore, Fig. 3d demonstrates the dependence of the absorption of the coupled-bar on the spacing (*S*). The absorption peaks all reach high values above 90%, which is 99%, especially when *S* is greater than 0.15 mm.

**Figure 4:**
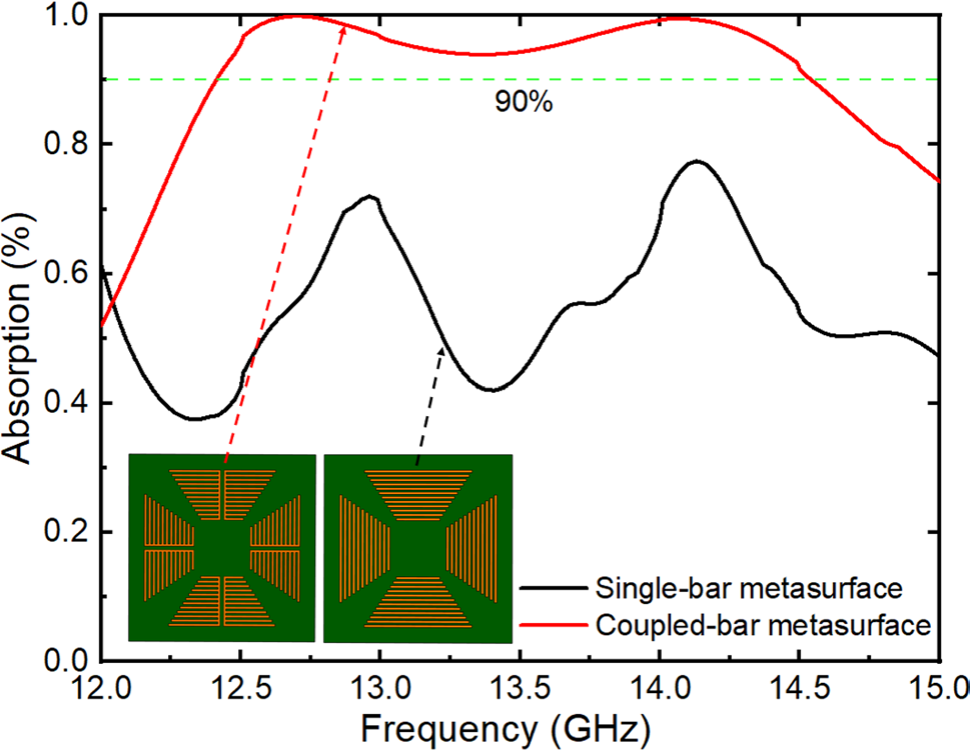
Compared absorption of the metasurfaces constructed with single and coupled-bars.

After performing comprehensive investigations on the absorption characteristics of single and coupled-bars, a metasurface was designed with the unit-cell parameters, as illustrated in Fig. 1b. The metasurface with single bars differed from that with coupled-bars in that it lacked a separation space in the middle and a connection between bars in the vertical direction. The unit cells of the two metasurface structures are depicted in the subfigure of Fig. 4. To simulate the absorption of the metasurface, a unit cell was calculated with periodic boundary conditions in two directions in the unit-cell plane. The unit-cell boundary conditions were chosen for the *x-* and *y*-directions in Computer Simulation Technology Microwave Studio (CST-MWS) software^37^. The absorption spectra of the two types of structures are shown in Fig. 4. For the single-bar structure (black curve), a broad absorption band was formed due to the superposition of neighboring metallic bars. However, as only 2/4 of the branches in the unit cell strongly interacted with the EM waves, the obtained absorption was not high. The absorption of the single-bar structure is observed to be between 40 and 60% in the frequency range from 12 to 15 GHz.

In contrast, for the coupled-bar structure (red curve), the absorption band is as wide as that for the single-bar structure, but the absorption is much greater. An absorption band above 90% is observed from 12.40 to 14.55 GHz. The minimum absorption in the band is also very high at 94%. The difference in absorption intensity between the two structures was attributed to the fact that the coupled-bar structure could work for different incident polarization angles, as discussed in Fig. 3a. All 4/4 branches of the unit cell with 8 coupled-bars in the metasurface responded to the EM waves, thereby leading to strong and broad absorption, similar to the interaction with external resistors in the structure.

**Figure 5:**
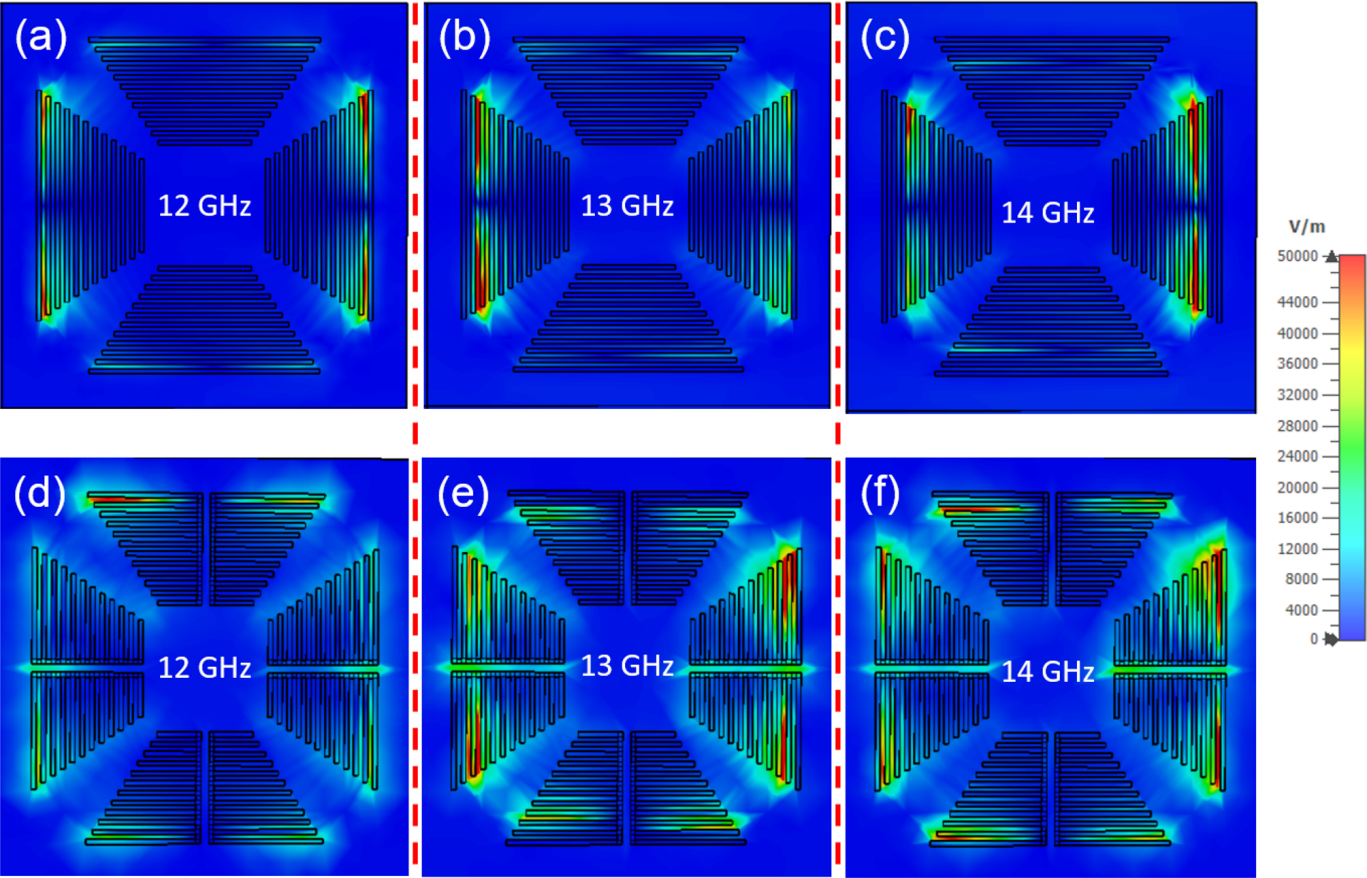
Comparison of the E-field intensity distributions in a unit cell of the structure constructed with (**a**–**c**) single- and (**d**–**f**) coupled-bars at various frequencies.

To better understand the high absorption in a wide frequency range of a metasurface with coupled-bars, detailed studies on the E-field distributions at various frequencies are necessary. Figure 5 shows the E-field intensity distributions in the unit cells of the two types of configurations at frequencies of 12, 13 and 14 GHz. For the single-bar configuration, when the sampling frequency changes from 12 to 14 GHz, a bar in the unit cell resonates for each sampling frequency, as shown in Fig. 5a–c. The resonant bar position in the unit cell tends to move inward as the frequency shifts from 12 to 13 to 14 GHz. This is reasonable because the bars with larger lengths resonate at lower frequencies. Due to the polarization of the incident EM wave, only the two branches on two sides of the unit cell are arranged vertically to respond to the EM wave. For the remaining two branches above and below, the response is nearly negligible. This is very different from the electric field distributions (Fig. 5d–f) in the coupled-bar configuration, where all four branches in the unit cell have resonance regions. Of course, the outer bars correspond to small frequencies, while the inner bars resonate at larger frequencies. In regard to the resonance intensity, the E-field strength is similar, but the resonance areas (8) for coupled-bars are greater than those (2) for single bars. At a frequency of 12 GHz, the coupled-bar unit cell shows that the corresponding E-field intensity is quite small, which is reasonable when examining the absorption spectrum presented in Fig. 4. The 12-GHz frequency region is outside the main absorption band of the coupled-bar case. In this frequency region, the single-bar configuration reveals slightly better absorption than the coupled-bar one. The results of the absorption spectrum and E-field distribution show that the coupled-bar configuration is suitable for designing a metasurface with high absorption in a wide frequency range without employing external electric components or special materials.

**Figure 6:**
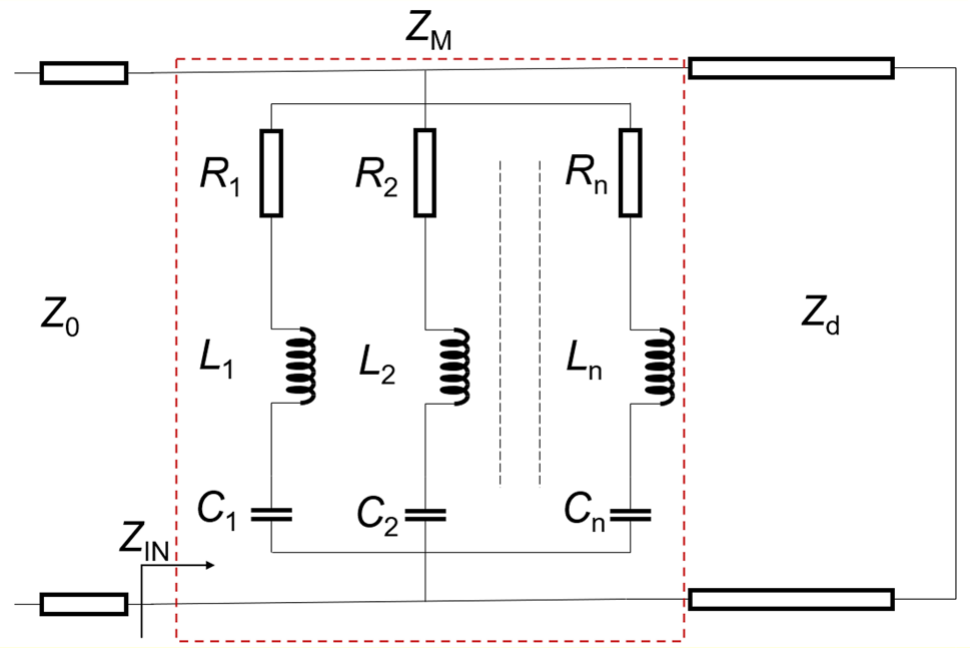
Equivalent circuit model of the proposed metasurface.

The proposed metasurface can be described by an equivalent circuit model in Fig. 6, by combining the transmission line theory^38,39^. The trapezoidal structure on the metasurface can be represented by the *RLC* series circuits. The metal ground on the back is modeled as a short circuit due to the reflecting property. The free space and dielectric layers are considered as transmission lines with impedance *Z*_0_ and *Z*_d_, respectively.

The impedance of the trapezoidal structure can be expressed as

1 $$ Z_{M} = \left( {R_{1} + j\omega L_{1} + \frac{1}{{j\omega C_{1} }}} \right)||\left( {R_{2} + j\omega L_{2} + \frac{1}{{j\omega C_{2} }}} \right)|| \cdots ||\left( {R_{n} + j\omega L_{n} + \frac{1}{{j\omega C_{n} }}} \right). $$

Hence, the input impedance of the metasurface can be derived to be

2 $$ Z_{IN} = \frac{{Z_{M} Z_{d} }}{{Z_{M} + Z_{d} }}. $$

According to the transmission line theory, the reflection coefficient of the metasurface can be calculated as follows;

3 $$ \Gamma = \frac{{Z_{IN} - Z_{0} }}{{Z_{IN} + Z_{0} }}. $$

Based on Eq. (3), to obtain a high absorption level (resulting in a low reflection coefficient) across a broad frequency spectrum, the input impedance (*Z*_IN_) should be close to the free-space impedance (*Z*_0_). In the trapezoidal structure, the resonance frequencies of the coupled-bars are in close proximity to each other, and the coupling effect between them is high. Consequently, the operational principle of the trapezoidal structure resembles that of a multilayer frequency-selective surface^40^, enabling the generation of a broad absorption band.

### Experimental Verification and Comparison

**Figure 7:**
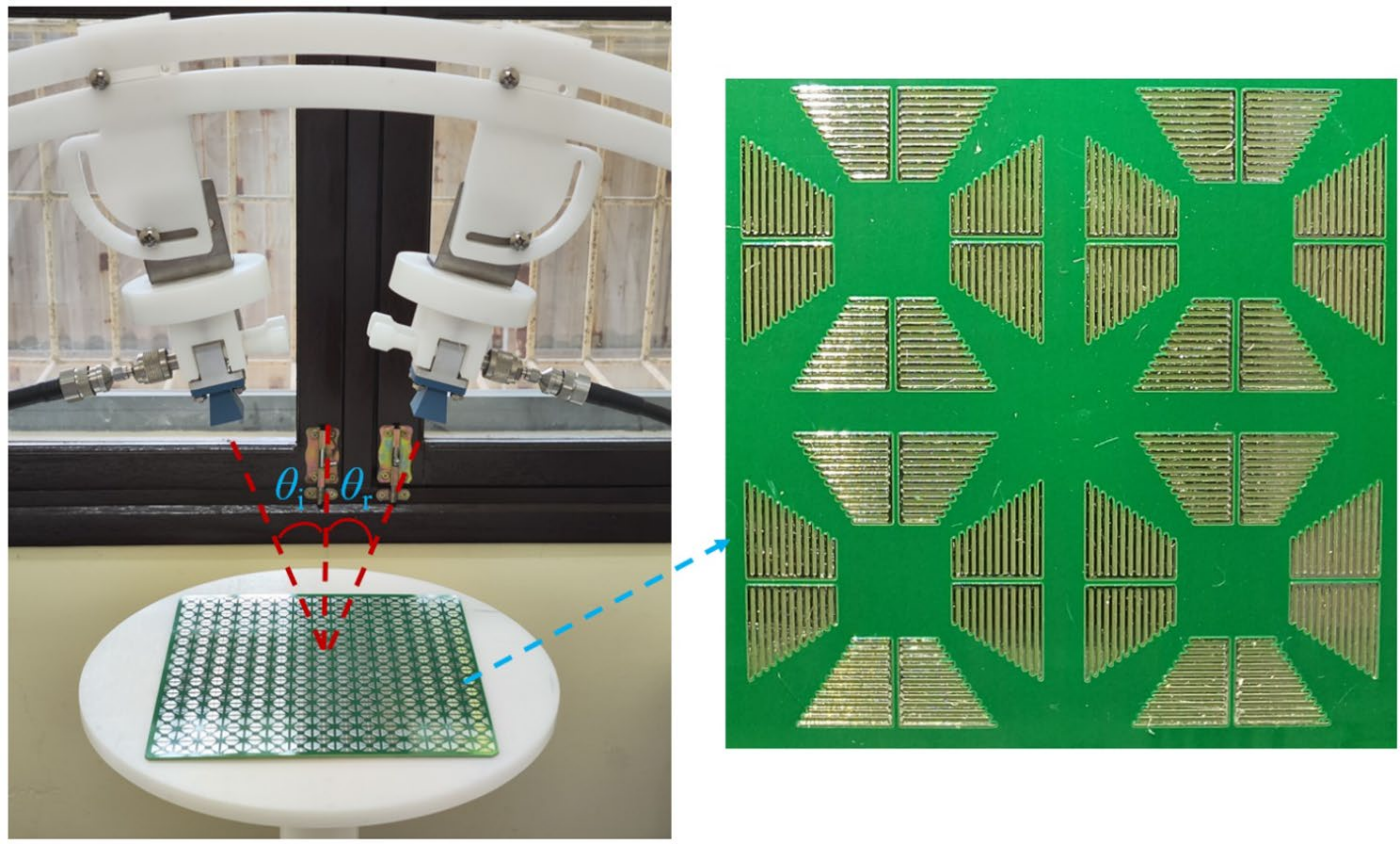
Experimental setup and the fabricated metasurface.

To validate the design and analyses in the aforementioned sections, we fabricated and measured the absorption characteristics of the metasurface^10,24,31^. The metasurface was constructed based on the unit cells depicted in Fig. 1b and had the following specific dimensions: *D* = 13, *P* = 1.1, *L* = 3.5, *W* = 0.15, *S* = 0.15, and *X* = 0.2 mm. The parameters *W* and *S* were chosen to be 0.15 mm because this was the easy limit of the manufacturing process. The simulation results indicated that, with smaller values of *W* and *S*, better absorption results were obtained. However, manufacturing at such a small size is a challenge in terms of cost. The metasurface was fabricated using standard PCB technology with an FR-4 substrate that was 2 mm in thickness. The thickness of the bottom Cu layer was 0.035 mm. The metasurface was constructed with 15 × 15 unit cells. The total dimensions were 200 × 200 mm, including the size of the edge. This size was large enough to obtain measurement results with small errors. The experimental setup is described in Fig. 7.

**Figure 8:**
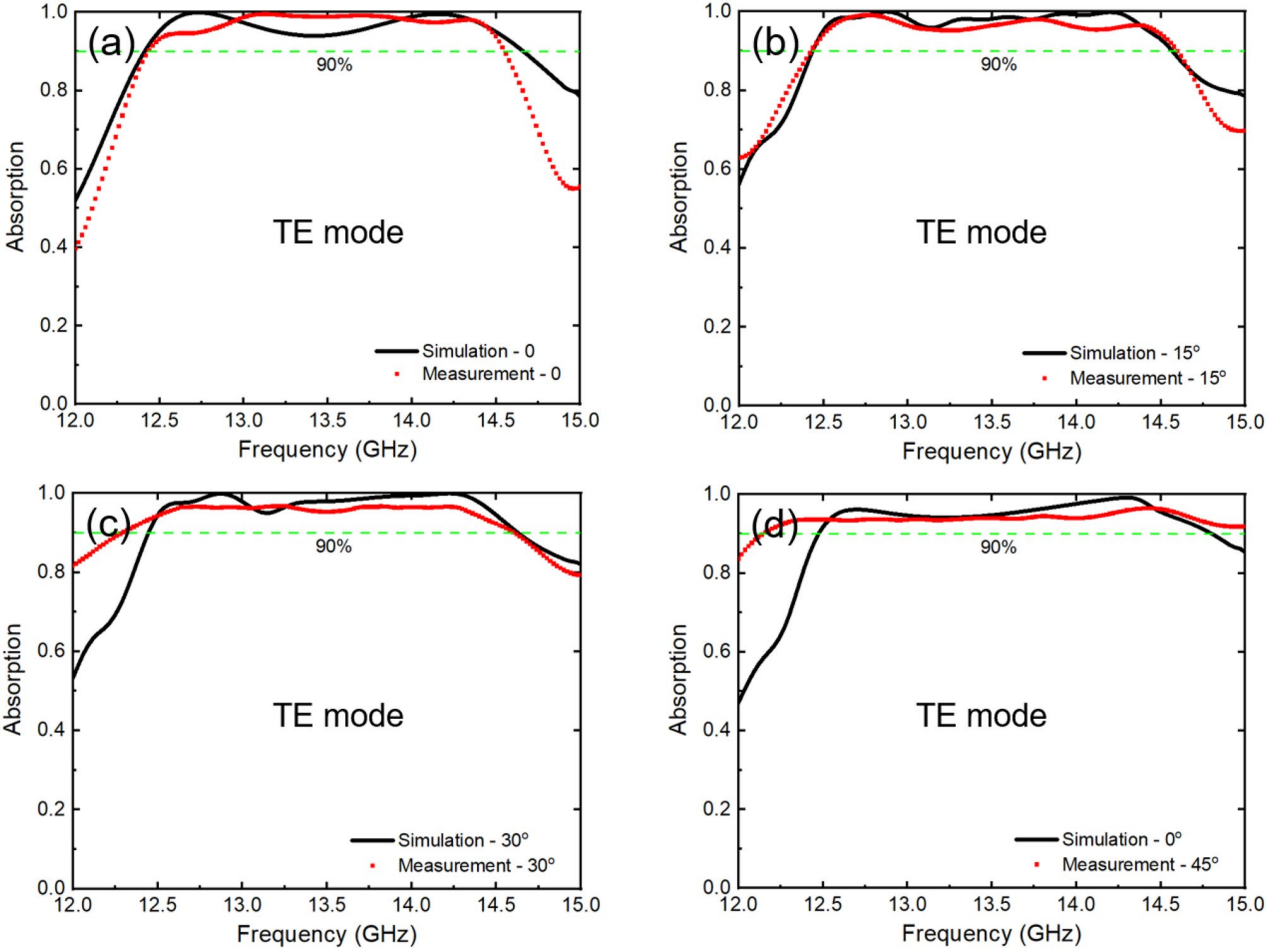
Comparison between the simulated and measured absorption of the metasurface in TE mode at (**a**) 0, (**b**) 15°, (**c**) 30° and (**d**) 45°.

The absorption characteristics of the metasurface were examined through both simulation and measurement at various incident angles ranging from 0 to 45 degrees, as shown in Fig. 8. The results reveal that the metasurface exhibits a wide absorption band above 90%, covering a frequency range of 12.40 to 14.55 GHz at all the investigated incident angles (0, 15, 30 and 45 degrees). Notably, the range for an absorption above 93% is also wide enough to be from 12.5 to 14.4 GHz. Nevertheless, slight disparities between the measurement and simulation results were observed due to the inherent errors associated with the manufacturing and measurement processes.

**Figure 9:**
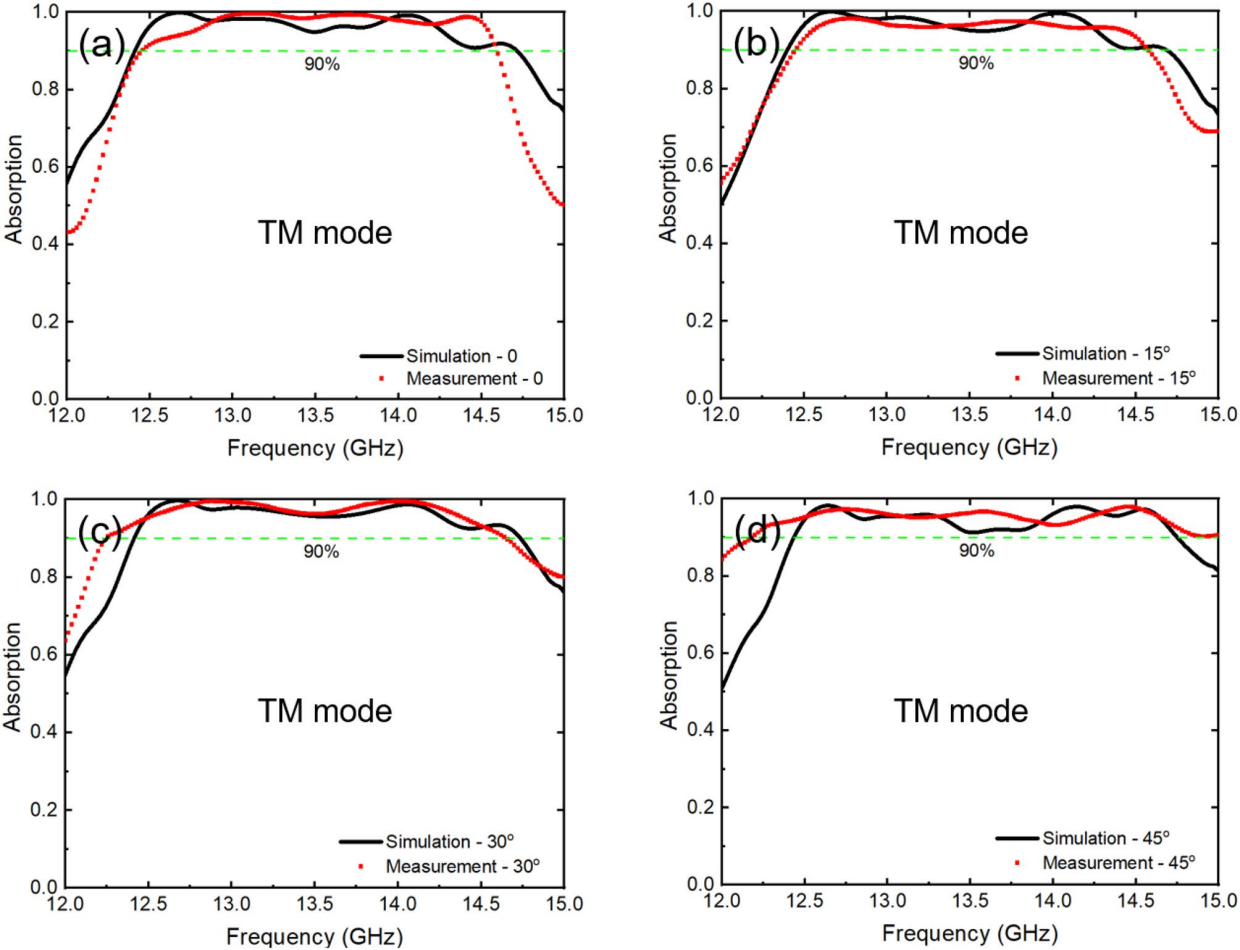
Comparison between simulated and measured absorption of the metasurface in the TM mode at (**a**) 0, (**b**) 15°, (**c**) 30°and (**d**) 45°.

Figure 9 shows the simulated and measured results of the absorption spectra of the metasurface when subjected to incident EM waves in the TM polarization, which are similar to those obtained with TE-polarized EM waves. The metasurface demonstrates an absorption above 90% in a wide frequency range from 12.5 to 14.6 GHz. Accordingly, the overall absorption spectra of the designed 2D metasurface exhibit high absorption over a wide range of frequencies and are insensitive to the polarization and incident angle of incident EM waves without external electric components or special materials.

## Discussion

Metamaterials for broadband absorption have been studied before, and there are also some notable results^29,31,32,34^. However, to achieve a wide absorption band, previous studies often used lumped elements^29^ or multilayered structures^31,34^. If, instead of the above configurations, only a metallic single-layer on a dielectric substrate^18,33^ is employed, it is challenging to achieve the broadband absorption. The detailed performance comparison of this work and previous ones are shown in Table 1. As we can see, the absorption bandwidth from 12.4 to 14.55 GHz is considered relatively wide with a high absorption, compared to other microwave absorbers. The absorption band turned out be wider with a structure by using lumped elements or multilayer. However, the manufacturing structure was very complex and expensive. At the same time, there were concerns about the stability and durability. Even for the single-layer structures that do not use lumped elements, the absorption band of our proposed metasurface is wide. For example, in Ref. 34, the absorption band was only from 13.40 to 14.25 GHz. We still continue to investigate and optimize the proposed structure to obtain even a wider absorption bandwidth.

**Table 1:** Comparison of the absorption performance among different metamaterial absorber configurations.

Ref	Bandwidth (GHz)	Absorption	Lumped elements/Mutilayer	Polarization (Both TE and TM?)	Incident angle
^18^	Peaks at 14.5, 16.5	> 90%	No/No	Both	0, 45°, 90°
^29^	4.68–13	> 80%	Yes/No	Both	0–15°
^31^	4.52–25.42	> 80%	Yes/Yes	Both	0–15°
^32^	9–15	> 80	Yes/No	Both	0–45°
^33^	11.21–11.49 13.92–14.85 17.66–17.87	> 90%	No/No	Both	0–45°
^34^	13.40–14.25	> 90%	No/Yes	Both	0–30°
This paper	12.4–14.55	> 90%	No/No	Both	0–45°

This study presents the design and fabrication of a metasurface that shows high absorption ability for EM waves. The metasurface unit cell comprises only metallic bars of varying lengths. No external electric components or special materials were employed. Elucidating the mechanism of superposition of the absorption peaks, we achieved a high (exceeding 90%) absorption band spanning a wide frequency range from 12.4 to 14.55 GHz. The absorption characteristics of the designed metasurface in this frequency range suggest its significant potential for various applications, including satellite communication, stealth radar, and radar surveillance. The simulation and experimental results have demonstrated the superior properties of metasurfaces, including wideband absorption, high absorption, 2D structure, insensitivity to polarization, and good response to incident angles up to 45 degrees. We have also presented a detailed analysis of the operating mechanism and design ideas of the metasurface. The metasurface was based on a simple design and fabricated by using easy and standard PCB technology with an inexpensive FR-4 layer between the metasurface structure and sealing bottom Cu layer. In comparison to other methods, such as the use of external electric components such as resistors or special materials or 3D multi-layer structures to develop a wideband absorption range, our approach offers advantages in terms of compactness, ease of fabrication, and cost-effectiveness.

## Methods

### Numerical simulation

All the simulation results were obtained by using a commercially available, finite-element-method solver-based EM simulator, CST-MWS software. To simulate the absorption of the metasurface, we calculated the unit cell with periodic boundary conditions in two directions of the metasurface plane. The E-field distribution was then achieved at frequencies within the operating range of the metasurface.

### Experimental configuration

The measurements were performed by using a Vector Network Analyzer (VNA) (Rohde & Schwarz ZNB20) with a pair of wideband antennas from 12 to 18 GHz. The two channels of the VNA were mounted with antennas to control the incident wave and analyze the received data. There, one channel was connected to the transmitting antenna, while the other was linked to the receiving antenna. To ensure proper measurement of the absorption performance under both normal and oblique incidence conditions, the two antennas were moved together for different incident angles so that the incidence angle *θ*_*i*_ and reflection angle *θ*_*r*_ were equal.

## Data Availability

The data that support the findings of this study are available from the corresponding author upon reasonable request.
